# Empowering grassroots innovation to accelerate biomedical research

**DOI:** 10.1371/journal.pbio.3001349

**Published:** 2021-08-09

**Authors:** Bastian Greshake Tzovaras, Michael Rera, Edwin H. Wintermute, Katharina Kloppenborg, Juliette Ferry-Danini, Guy Aidelberg, Rachel Aronoff, Ariel Lindner, Dusan Misevic

**Affiliations:** 1 INSERM U1284, Université de Paris, Center for Research and Interdisciplinarity, Paris, France; 2 Centre for Health Ethics Law and Development, University of Toronto, Canada; 3 Association Hackuarium, Lausanne, Switzerland

## Abstract

The purpose of biomedicine is to serve society, yet its hierarchical and closed structure excludes many citizens from the process of innovation. We propose a collection of reforms to better integrate citizens within the research community, reimagining biomedicine as more participatory, inclusive, and responsive to societal needs.

Publisher’s note: This Perspective is one of the 2 winning essays of the “Reimagine Biomedical Research for a Healthier Future Essay Challenge,” launched by the Health Research Alliance (HRA) in partnership with the Public Library of Science (PLOS). This publication is coordinated with that of the other winning essay in *PLOS Medicine*. The competition was intended to spark a discussion around the future of biomedical research; publication does not imply endorsement from HRA or PLOS.

Biomedicine today is organized from the top down. National and supranational policymakers set priorities for funding agencies, which, in turn, distribute money to institutions and individual scientists. These organizational choices are motivated, in part, by the complexity and uncertainty of the biomedical research enterprise. However, institutional hierarchies come at a cost: openness and responsiveness. Large structures become incomprehensible and closed to anyone looking in from the outside, while also slowing down research due to a lack of reactivity in the face of emerging priorities. The result is that patients’ needs can go unheard within institutions whose very mission is to hear them—a dire, but fixable, problem.

In contrast, during the Coronavirus Disease 2019 (COVID-19) pandemic, we saw many examples of grassroots responsiveness. When larger institutions failed to provide personal protective equipment, fablabs and makerspaces around the world self-organized to produce and distribute masks and face shields to both healthcare workers and the public at large [1]. In a similar spirit, biohackers and do-it-yourself (DIY) biology teams independently developed and deployed rapid COVID-19 tests. Within months, the first tests were rolled out in countries like Sri Lanka, with limited access to conventional biomedical institutions.

Grassroots innovation also represents a strategy to address “undone science”—typically unfunded or ignored research that is deemed important by civil society and social movements [2]. An emblematic case is the Open Drug Discovery (ODD) movement [3], which aims to discover drug candidates for neglected diseases such as tuberculosis or trypanosomiasis. There are common reasons for this institutional neglect: These diseases are not prevalent in rich countries, where public funding priorities are set, or are not profitable to pharmaceutical companies, where private funding priorities are set. However, they motivate many individual researchers and patient activists. The ODD movement makes use of a distributed network of open partners that share knowledge and materials to innovate without the need for top-down organization [4]. The field of participant/patient-led research represents another bottom-up solution for “undone science” in cases where traditional research does not address the needs articulated by the patients. For example, while diabetes research is well funded, only a fraction of that spending is seen by patients as improving their quality of life, so they took the initiative to self-organize such research in the #WeAreNotWaiting movement. Their achievements, including the production of a fully operational DIY artificial pancreas built from open-source hardware and software [5], showcase the potential sophistication of grassroots innovation.

Despite these compelling cases for bottom-up innovation, many barriers still prevent its adoption on a larger scale—successful examples remain the exception to the rule. The main barrier faced by community-led movements is access. The open science movement has made substantial progress in improving access to research data and publications, but other research essentials remain inaccessible to would-be biomedical innovators who act outside of institutions. We should seek to improve access to hardware, lab equipment, consumables, and funding, as well as to ethical and biosafety oversight (Fig 1).

**Fig 1:**
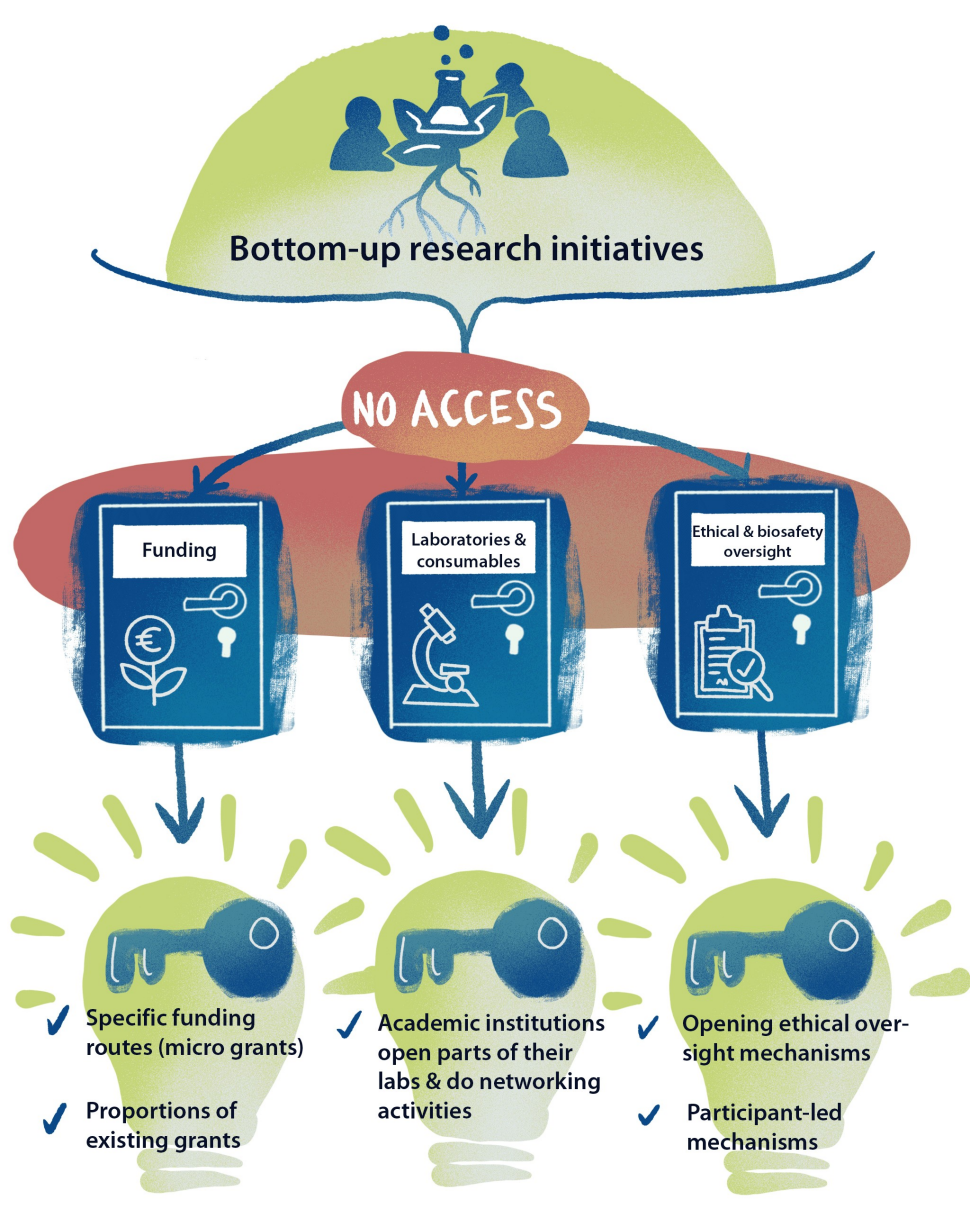
Barriers and solutions for bottom-up research initiatives. We identify 3 types of barriers: access to funding, access to laboratories and consumables, and access to ethical and biosafety oversight. For each, we offer actionable solutions.

Biomedicine is increasingly reliant on expensive technology. Laboratory consumables can often be purchased only through research institutions and recognized corporations, even when they do not present biosafety concerns. While grassroots labs might buy some technology secondhand, the lack of even 1 reagent in a complex protocol is enough to scuttle an entire project. Grassroots innovators must resort to work-arounds through personal relationships with professional researchers. This ad hoc mentorship, while admirable, is not scalable.

Necessity being the mother of invention, “grassroots innovation” often means “frugal innovation,” but remains far from free. Traditional funding channels, including government and nonprofit granting agencies, often exclude extra-institutional groups from competing for funding. While larger bottom-up projects may solve this by incorporating or seeking other forms of legal status, this incurs a significant bureaucratic overhead and leads to consequential innovation delays.

Grassroots innovators also need access to ethical oversight and training to understand the rules of safe and ethical research. The structure and mission of institutional review boards (IRBs) varies by country, but rarely do they recognize noninstitutional projects. Grassroots teams that wish to perform ethical research, therefore, have no way to get an independent opinion and feedback. This is particularly problematic when grassroots innovators engage in self-experimentation, leading to ethical and legal gray areas not confronted in traditional clinical research.

To lift these barriers, we call for 3 specific actions from academic institutions and funders (Fig 1):

1. **Expand networks and offer official status:** Community bio labs exist and thrive around the world—in places like Oakland, Lima, Paris, and Hong Kong—but many communities remain underserved. To scale up access, academic institutions should open parts of their lab spaces for grassroots innovation teams and projects. A formal legal status of citizen researcher should be created to unlock legal protections and workplace insurance. This status could follow a registered internship model, including proper training in lab safety, ethical research conduct, and mentorship by resident researchers. While traditional academic institutions are the primary target for such programs, these initiatives could be expanded to incubator lab spaces. Community mentorship should be rewarded when evaluating research impacts, adding further incentives for researchers.
2. **Develop microgrants targeted to grassroots groups:** Funders should create specific funding routes that are open to community projects. Importantly, the grants—which do not need to be large—should account for the fact that these projects are typically not yet organized in a fixed legal entity. Successful examples of this include the Prototype Funds of Germany and Switzerland, which support open-source projects advancing civic tech and data literacy [6,7], and Just One Giant Lab, a French nonprofit platform that awarded nearly 50 microgrants to grassroots pandemic response projects [8]. Funding agencies should also allocate a portion of classical research grants to bottom-up partnerships, facilitating mentoring relationships with institutional researchers. These relationships can be mutually beneficial: In addition to supporting grassroots projects, they can help to improve traditional research, e.g., by informing study designs and enrollment procedures, thus improving their efficiency and impact.
3. **Explore alternative mechanisms of ethical and biosafety oversight:** Although bottom-up innovators are expected to follow the same ethical, safety, and scientific standards as traditional research, the mechanisms to ensure this need not be the same. While a simple first step is to open up ethical review procedures to bottom-up innovators, it should not be the last. Alternative models should be explored, including participant-led ethics committees and crowdsourced review in the model of open software. We encourage active participation by funders and researchers in the development of innovative review mechanisms [9].

Institutional inertia can make change seem impossible, yet biomedical research must change. There is too much “undone science,” too many missed opportunities, and too many patient needs unmet. Citizens are already reimagining biomedicine by taking an active role in urgently needed research. Institutions that embrace, welcome, and support public participation will help unlock the full potential of grassroots innovation.

## Abbreviations

COVID-19: Coronavirus Disease 2019
DIY: do-it-yourself
IRB: institutional review board
ODD: Open Drug Discovery
